# Extracellular cathepsin S and intracellular caspase 1 activation are surrogate biomarkers of particulate-induced lysosomal disruption in macrophages

**DOI:** 10.1186/s12989-016-0129-5

**Published:** 2016-04-23

**Authors:** Caroline S. Hughes, Liza M. Colhoun, Baljinder K. Bains, Joanne D. Kilgour, Roberta E. Burden, James F. Burrows, Ed C. Lavelle, Brendan F. Gilmore, Christopher J. Scott

**Affiliations:** 1 Molecular Therapeutics, School of Pharmacy, Queen’s University Belfast, 97 Lisburn Road, Belfast, BT9 7BL UK; 2 Centre for Experimental Medicine, Queen’s University Belfast, Belfast, BT12 6BA UK; 3 Innovative Medicines and Early Development, Drug Safety and Metabolism, Regulatory Safety, AstraZeneca, Mereside, Alderley Park, Macclesfield, Cheshire, SK10 4TG UK; 4 Adjuvant Research Group, School of Biochemistry and Immunology, Trinity Biomedical Sciences Institute, Trinity College Dublin, Dublin 2, D02 PN40 Ireland

**Keywords:** Nanoparticles, Toxicity, Cathepsin S, Caspase 1, IL-1β, Affinity-binding probes, Proteolytic activity

## Abstract

**Background:**

Particulate matter has been shown to stimulate the innate immune system and induce acute inflammation. Therefore, while nanotechnology has the potential to provide therapeutic formulations with improved efficacy, there are concerns such pharmaceutical preparations could induce unwanted inflammatory side effects. Accordingly, we aim to examine the utility of using the proteolytic activity signatures of cysteine proteases, caspase 1 and cathepsin S (CTSS), as biomarkers to assess particulate-induced inflammation.

**Methods:**

Primary peritoneal macrophages and bone marrow-derived macrophages from C57BL/6 mice and *ctss* ^*−/−*^ mice were exposed to micro- and nanoparticulates and also the lysosomotropic agent, L-leucyl-L-leucine methyl ester (LLOME). ELISA and immunoblot analyses were used to measure the IL-1β response in cells, generated by lysosomal rupture. Affinity-binding probes (ABPs), which irreversibly bind to the active site thiol of cysteine proteases, were then used to detect active caspase 1 and CTSS following lysosomal rupture. Reporter substrates were also used to quantify the proteolytic activity of these enzymes, as measured by substrate turnover.

**Results:**

We demonstrate that exposure to silica, alum and polystyrene particulates induces IL-1β release from macrophages, through lysosomal destabilization. IL-1β secretion positively correlated with an increase in the proteolytic activity signatures of intracellular caspase 1 and extracellular CTSS, which were detected using ABPs and reporter substrates. Interestingly IL-1β release was significantly reduced in primary macrophages from *ctss* ^*−/−*^ mice.

**Conclusions:**

This study supports the emerging significance of CTSS as a regulator of the innate immune response, highlighting its role in regulating IL-1β release. Crucially, the results demonstrate the utility of intracellular caspase 1 and extracellular CTSS proteolytic activities as surrogate biomarkers of lysosomal rupture and acute inflammation. In the future, activity-based detection of these enzymes may prove useful for the real-time assessment of particle-induced inflammation and toxicity assessment during the development of nanotherapeutics.

**Electronic supplementary material:**

The online version of this article (doi:10.1186/s12989-016-0129-5) contains supplementary material, which is available to authorized users.

## Background

Non-biodegradable particulate matter, such as silica and asbestos, has been shown to stimulate the innate immune system and cause acute inflammation [1]. Accumulation of particulates in the airways from chronic exposure leads to cell injury, silicosis and progressive lung fibrosis, and is associated with autoimmunity, lung cancer and cardiopulmonary failure [2]. Accordingly, while rapid progress is being made in the application of nanotechnology in therapeutics, primarily for medical diagnostics and drug delivery, there are concerns that such pharmaceutical preparations could induce the same unwanted inflammatory and toxic side effects as incidental particulate matter. Therefore, dissecting the common mechanisms which underlie particle-induced inflammation warrants further investigation and identifying biomarkers to monitor the initial stages of this process may prove useful.

Once in the circulation, particulate matter can be ingested by resident macrophages inducing a pro-inflammatory phenotype that if unchecked or prolonged, can inadvertently induce tissue damage and fibrosis [3]. A key early proinflammatory cytokine released from macrophages is interleukin 1 beta (IL-1β). Normally under stringent control in physiological conditions, IL-1β dysregulation has been shown to underlie a diverse range of diseases including Alzheimer’s disease, silicosis and rheumatoid arthritis [4, 5].

Post-translational processing of pro-IL-1β into its biologically active form is facilitated by the formation of a multi-molecular complex termed the inflammasome, which acts as a platform to drive the autocatalytic processing and activation of pro-caspase 1, which subsequently cleaves pro- IL-1β to mature IL-1β, which is then secreted [6, 7]. The assembly of the inflammasome requires activation of a cytosolic, danger-sensing Nod-like receptor (NLR) in response to inflammatory stimuli or cellular stress. The most extensively studied NLR, NLRP3, is activated in response to a variety of crystalline and particulate matter, including silica, alum, monosodium urate crystals, cholesterol crystals and amyloid fibres [8–12]. As many particulates trigger lysosomal rupture following phagocytosis by macrophages, a relationship between lysosomal destabilisation and NLRP3 inflammasome activation has been established and is thought to be mediated by leakage of lysosomal cathepsins [13–15].

The increasing appreciation for the roles of proteases, such as cathepsins, in pathological conditions has led to much interest in their prognostic and diagnostic potential [16]. Thus, while levels of IL-1β can be easily determined using ELISA or associated technologies, we hypothesized that the measurement of caspase 1 and cathepsin activities may constitute alternative, more useful real-time readouts of lysosomal disruption and particle-induced acute inflammation. Furthermore, using activity-based proteomic detection methods, proteolytic activities may prove more translatable to in vivo imaging applications, which could assess the potential toxicity of particulates directly and in real time [17].

In this current study, we examine the potential of using caspase 1 and cathepsin S (CTSS) activation as a readout of lysosomal disruption and acute inflammation. We report that CTSS activity may represent an extracellular marker of particle-induced inflammation as opposed to intracellular caspase 1 activation. Furthermore, we demonstrate that that CTSS plays a significant role in IL-1β secretion from particulate-treated macrophages, highlighting an unappreciated role for CTSS in the innate immune response.

## Results

### Particulates induce the release of proinflammatory mediator IL-1β in both peritoneal and bone marrow derived macrophages

**Fig. 1:**
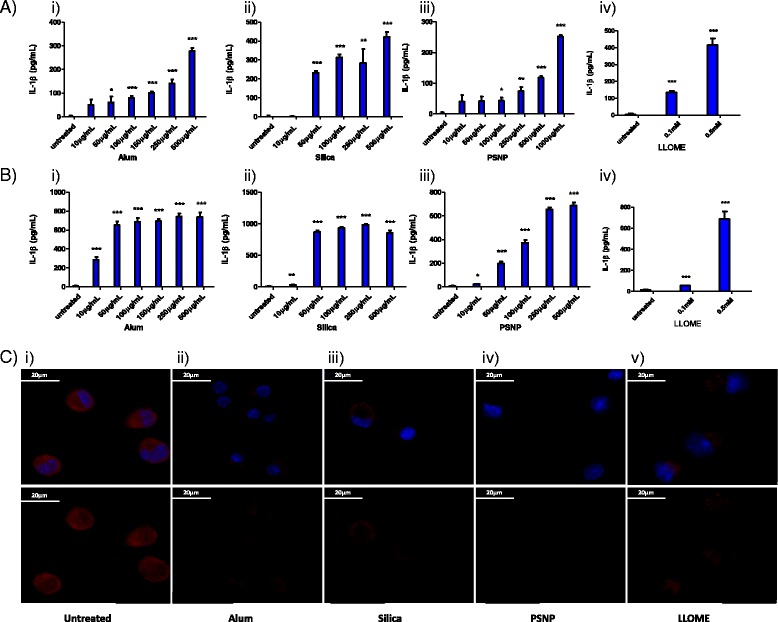
Particulates and LLOME induce perturbations in lysosomal compartments leading to the release of IL-1β. ELISA analysis of IL-1β production in supernatants harvested from: **a** Peritoneal macrophages primed for 3 h with LPS (100 ng/mL) and stimulated for 16 h with a concentration range (10–1000 μg/mL, 22–2200 pg/cell) of i) alum, ii) silica, iii) PSNP, iv) LLOME, **b** BMDMs primed for 3 h with LPS (100 ng/mL) and stimulated for 16 h with a concentration range (10–1000 μg/mL, 22–2200 pg/cell) of i) alum, ii) silica, iii) PSNP, iv) LLOME. For all experiments *n* = 4 +/− standard error. **c** Confocal microscopy of acidified lysosomes labelled with acridine orange (3 μM) in peritoneal macrophages which were i) left untreated or treated for 4 h with ii) 500 μg/mL alum, iii)250 μg/mL silica, iv) 500 μg/mL PSNP, v) 0.5 mM LLOME. Top panel: nuclear dapi stain (*blue*) + acridine orange (*red*). Lower panel: acridine orange only. Images representative of 2 independent experiments., scale bar = 20 μm

In our initial experiments we validated that particulate matter had the ability to induce the release of IL-1β as previously described [11, 13]. We examined the potential of different particle compositions to activate IL-1β responses in macrophages which were primed with toll-like receptor ligand, LPS to induce the initial expression of pro- IL-1β. We found that a range of non-biodegradable particulates including alum microparticles, 3–10 μm in diameter, and silica and polystyrene nanoparticles (PSNP), both 100 nm in diameter, induced the release of IL-1β from both LPS-primed peritoneal and bone marrow derived macrophages (BMDMs), in a dose-dependent manner (Fig. 1a and b respectively). Furthermore, we were able to mimic the effect of particulates on IL-1β release through the treatment of macrophages with the lysosomotropic agent, L-leucyl-L-leucine methyl ester (LLOME) (Fig. 1a(iv) and b(iv)). LLOME acts as a detergent which accumulates in acidic vesicles, inducing lysosomal rupture and has been previously employed as a surrogate reagent to analyse particle-induced inflammation [14]. Interestingly, no measurable release of IL-1β from the cells was observed upon co-incubation with comparable concentrations of biodegradable 150 nm poly-lactide-co-glycolic acid (PLGA) nanoparticles (Additional file 1: Figure S1). To ascertain if the particles were affecting lysosomal integrity, lysosomes in peritoneal macrophages were stained with acridine orange and visualised using microscopy (Fig. 1c). In the untreated cells, acridine orange accumulated in acidic lysosomal compartments which can be seen as highly concentrated red fluorescence in the cytosol, in a punctate pattern. However, treatment with particulates led to a dramatic reduction in red fluorescence in the cells, indicative of loss of lysosomal integrity and lysosomal rupture [18–20]. Staining of lysosomes following treatment of peritoneal macrophages with LLOME showed a similar effect on lysosomal staining (Fig. 1c). Taken together, these results confirm that non-biodegradable particulates induce the release of IL-1β in macrophage populations through destabilisation of lysosomal compartments, and we also establish that LLOME can be employed to replicate this effect in vitro.

### Caspase 1 activity can be visualised upon particle-induced lysosomal disruption

**Fig. 2:**
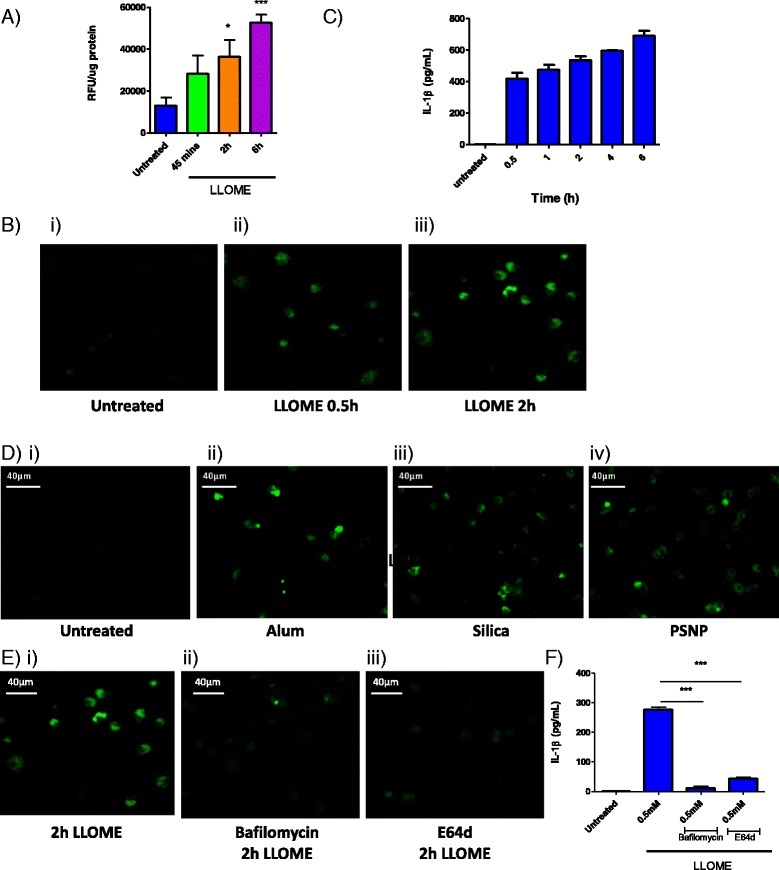
Caspase 1 is act ivat ed following LLOME and part icl e-induced lysosomal rupture in macrophages. **a** Caspase 1 act ivity in lysat es from LPS-pr imed BMDMs, tr eated with LLOME (0.5 mM) for 0.5 h to 6 h. Act ivity was measured using r eporter substrate Z-YVAD-AMC (60 μM), which upon hydrolysis by caspase 1produces a det ectabl e shift in RFU, n = 4 +/− standard error. **b** Confocal microscopy visual izing act ive caspase 1 in per itoneal macrophages i) l eft untreated or ii) treated with LLOME (0.5 mM) for 0.5 h or iii) 2 h. Cell s wer e incubat ed with the affinity-based FLICA probe, FAM-FLICA-FMK for 1 h, pr ior to washing and fixat ion. Images repr esentat ive of 3 independent exper iment s, scal e bar = 40 μm. **c** ELISA analysis of IL-1β released over t ime from LPS-pr imed BMDMs following treatment with LLOME (0.5 mM) for 0.5 h to 6 h, n = 4 +/standard error. **d** Confocal microscopy visual izing act ive caspase 1 in macrophages i) l eft untreated or treated for 2 h with ii) 500 μg/mL alum, iii)250 μg/mL sil ica , iv) 500 μg/mL PSNP. **e** Confocal microscopy visual izing act ive caspase 1 in macrophages i) treated with LLOME (0.5 mM) for 2 h or ii) pr e-treated with bafilomycin (250 nM) or iii) E64d (50 μM) pr ior to treatment with LLOME (0.5 mM) for 2 h. **f** ELISA analysis of IL-1β r el ease from LPSpr imed per itoneal macrophages pr e-treated with bafilomycin (250 nM) or E64d (50 μM) pr ior to treatment with LLOME (0.5 mM) for 6 h, n = 3 +/− standard error

IL-1β is produced and stored as a precursor (pro-IL-1β) that must be activated by the cytoplasmic cysteine protease, caspase 1, prior to secretion. Caspase 1 is produced as an inactive zymogen and requires proteolytic activation by the inflammasome. Caspase 1 activity in cell lysates can be selectively detected using the 7-Amino-4-methylcoumarin (AMC)-labelled fluorogenic peptidyl reporter substrate, Z-Tyr-Val-Ala-Asp-AMC. Upon proteolytic cleavage of the peptidyl sequence by caspase 1, a detectable shift in the emission spectrum of the fluorophore is produced, which acts as a readout of caspase 1 activity. Using LLOME as a model inducer of lysosomal rupture, it was demonstrated that stimulation of BMDMs led to an increase in cytoplasmic caspase 1 activity levels, which was apparent within 0.5 h and increased up to 6 h (Fig. 2a). In addition to using fluorogenic substrates, proteolytic activity can also be visualized using ABPs which act as suicide substrates, that become irreversibly attached to the active site of functional proteases, such as caspases and cysteine cathepsins, through nucleophilic attack of their highly active site thiol group, therefore allowing target proteases to be labelled [21, 22]. Accordingly, the application of a caspase 1-selective fluorescent probe, FAM-Tyr-Val-Ala-Asp-FMK, to detect and visualize the protease in macrophage cultures was evaluated. Control cells not treated with LLOME elicited no green fluorescent staining (Fig. 2b), however, upon treatment of the cells with LLOME, a clear cytoplasmic signal was observed from 0.5 h, which further increased at 2 h post-treatment, indicative of caspase 1 activity (Fig. 2b). Both readouts of increasing caspase activation over time, following LLOME treatment, were consistent with increased IL-1β secretion at the equivalent concentration and at comparable time points, confirming the utility of caspase 1 activity as a biomarker of lysosomal rupture (Fig. 2c). Finally, using the caspase 1-selective probe, caspase 1 activity was visualised in peritoneal macrophages which had been treated for 2 h with alum, silica and PSNP. Compared to the untreated cells, a clear increase in cytoplasmic labelling of active caspase 1 was visible in the particle-treated cells, as seen by increased green fluorescence (Fig. 2d). Taken together, these results validate the approach of measuring and visualizing intracellular caspase 1 activity as a surrogate biomarker for particle-induced lysosomal disruption and IL-1β release.

### Inhibition of lysosomal acidification or inhibition of cysteine cathepsins blocks caspase 1 mediated IL-1β release

During the course of the experiments to detect caspase 1 activity, we also examined the role of lysosomal proteases in the modulation of caspase 1 activity. Prior to treatment with LLOME, we incubated cells with the H(^+^)-ATPase proton pump inhibitor, bafilomycin A1, to abolish lysosomal acidification and thus cathepsin maturation and activation, showing that this reduced activation of caspase 1 (Fig. 2e). Similar depletion of caspase 1 activity was observed through pre-incubation of cells with the cell permeable, pan cysteine cathepsin inhibitor, E64d (Fig. 2e(iii)). In agreement with these effects on caspase 1 activity, it was also found that inhibition of the lysosomal proteases led to a significant decrease in the release of IL-1β, clearly highlighting the role of cysteine cathepsin activity in the control of caspase 1 activity (Fig. 2f).

### Particle-induced lysosomal disruption induces release of CTSS, which can be detected extracellularly

**Fig. 3:**
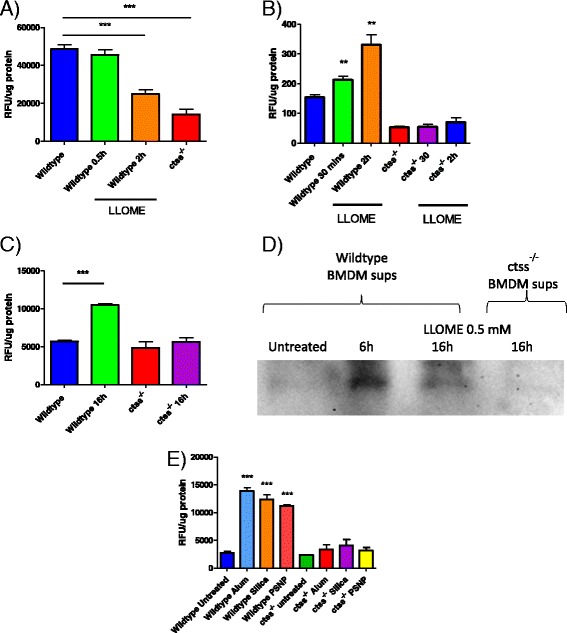
LLOME and particle-induced lysosomal rupture causes the release of active CTSS from macrophages. **a** CTSS activity in lysates measured as increased RFU, produced from the proteolytic cleavage of the reporter substrate, Z-VVR-AMC (50 μM). Lysates were generated from BMDMs which were left untreated or treated with LLOME (0.5 mM) for the indicated times. *Ctss* ^*−/−*^ cells were used as a control to determine background levels of substrate turnover not due to CTSS, *n* = 3 +/− standard error. **b** Extracellular turnover of Z-VVR-AMC (50 μM) by CTSS in supernatants harvested from BMDMs treated with LLOME (0.5 mM) for 0.5-2 h, *n* = 4 +/− standard error. **c** Extracellular turnover of Z-VVR-AMC (50 μM) by CTSS in supernatants harvested from BMDMs treated with LLOME (0. 5 mM) for 16 h, *n* = 3 +/− standard error. **d** Cell supernatants were incubated with biotin-PEG-LVG-DMK (10 uM) to label secreted mature CTSS from BMDMs treated with LLOME (0.5 mM) for 6 and 16 h. Supernatants from *ctss* ^*−/−*^ cells which had also been treated with LLOME (0.5 mM) for 16 h were used as a control to confirm the identity of labeled CTSS. Blot is representative of 3 independent experiments. **e** Extracellular turnover of Z-VVR-AMC (50 μM) by CTSS in supernatants harvested from BMDMs treated for 4 h with alum (500 μg/mL), silica (250 μg/mL) or PSNPs (500 μg/mL), *n* = 3 +/− standard error

A predominant cathepsin found in macrophages and up-regulated by pro-inflammatory stimuli is CTSS. We therefore asked what specific effect lysosomal disruption would have on its activity levels. As with caspases, cysteine cathepsins are also produced as inactive precursor zymogens and therefore real-time assessment of activity is more useful than analysis of protein levels alone. However, CTSS has broader substrate selectivity than caspase 1; thus in order to determine its activity levels accurately, selective conditions and substrates are required. The tripeptide substrate Z-Val-Val-Arg-AMC assayed at neutral pH can discriminate CTSS over closely related cathepsins that have inferior stability at neutralized conditions [23]. Using this substrate, we first examined intracellular CTSS-like activity levels in LPS-primed BMDMs, observing a higher specific activity in wild type lysates when compared to *ctss* ^*−/−*^ lysates, highlighting the stringency of the assay conditions in specifically measuring CTSS activity (Fig. 3a). Interestingly, following treatment of the cells with LLOME for a 2 h time point, the levels of CTSS-like activity were reduced in these lysates. As CTSS can remain active at cytosolic pH, the observed time-dependent inactivation of this protease by LLOME treatment may reflect the gradual inhibition of CTSS by endogenous inhibitors, such as cystatins, or potentially the secretion of this protease from the cell. As the prognostic and diagnostic value of CTSS in serum levels has previously been demonstrated for pathologies such as diabetes, atherosclerosis and aortic aneurysm [24–26], we also examined CTSS secretion from these cells by measuring activities in the corresponding supernatants. Here it was found that levels of CTSS activity rapidly increased at 0.5 h, following incubation of the cells with LLOME (Fig. 3b). Furthermore, supernatants from cells treated with LLOME for a longer 16 h time point demonstrated that CTSS activity persists extracellularly highlighting its potential as a biomarker (Fig. 3c).

ABPs have also been developed for detection of active CTSS. Incubation of the macrophage culture supernatants with the ABP, Biotin-PEG-Leu-Val-Arg-diazoketone, permitted chemiluminescent detection of active CTSS species via streptavidin-HRP conjugation to the bound biotin reporter group. In agreement with the results using the fluorescently labeled substrate, detection of active extracellular CTSS was only observed in the LLOME-treated cells and was detectable at both 6 and 16 h post-treatment; with no CTSS labeling observed in LLOME-treated *ctss* ^*−/−*^ supernatants (Fig. 3d). Next, we confirmed that treatment of macrophages with particulate matter had the same effect on CTSS secretion. Treatment of BMDMs with alum, silica or PSNP for 4 h, resulted in an increase in CTSS activity in cell supernatants, as measured by increased turnover of the reporter substrate Z-Val-Val-Arg-AMC (Fig. 3e). Taken together, these results clearly demonstrate that extracellular CTSS activity levels may also represent a useful biomarker of lysosomal disruption and particle-induced inflammation in macrophages and is detectable through use of substrate and ABP technologies.

### IL-1β release is reduced in LLOME and particle treated ctss^−/−^ macrophages

**Fig. 4:**
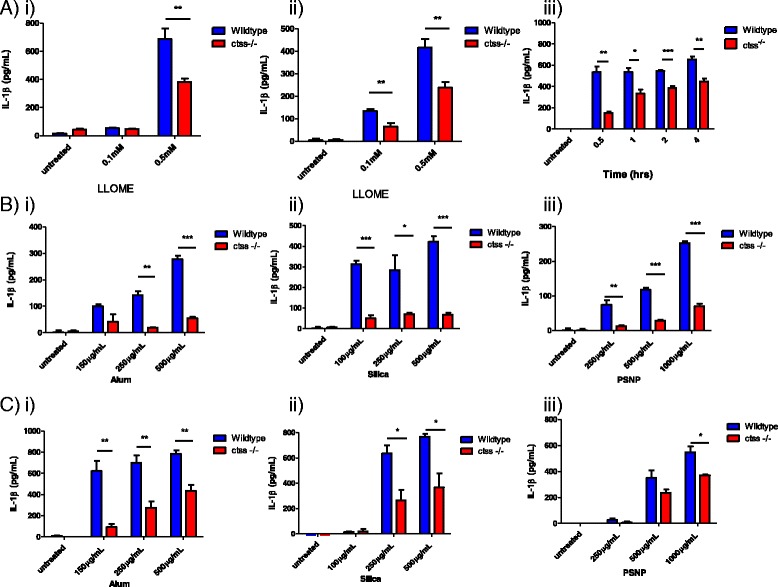
IL-1β release is reduced in LLOME and particle treated *ctss* ^*−/−*^ macrophages. **a** ELISA analysis of secreted IL-1β levels in supernatants from wildtype and *ctss* ^*−/−*^ i) LPS-primed peritoneal macrophages and ii) LPS-primed BMDMs which were treated for 16 h with LLOME (0.5 mM), *n* = 4 +/− standard error. iii) ELISA analysis of secreted IL-1β levels in supernatants from wildtype and *ctss* ^*−/−*^ BMDMs which were primed with LPS (100 ng/mL) prior to stimulation with LLOME (0.5 mM). Supernatants were harvested at timed intervals from 0.5 h to 4 h, *n* = 3 +/− standard error. **b** ELISA analysis of secreted IL-1β levels in supernatants from wildtype and *ctss* ^*−/−*^ LPS-primed peritoneal macrophages which were treated for 16 h with a concentration range of i) alum, ii) silica, iii) PSNP, *n* = 4 +/− standard error. **c** ELISA analysis of secreted IL-1β levels in supernatants from wildtype and *ctss* ^*−/−*^ LPS-primed BMDMs which were treated for 16 h with a concentration range of i) alum, ii) silica, iii) PSNP, *n* = 4 +/− standard error

Next, the functional significance of CTSS on IL-1β release in particulate-treated macrophages was assessed. Using both wild type and *ctss* ^*−/−*^ peritoneal and BMDMs, it was shown that LLOME treatment produced significantly reduced levels of IL-1β in CTSS deficient cells (Fig. 4a). In a time-course experiment in BMDMs treated with 0.5 mM LLOME, we observed that the reduction in IL-1β release in the *ctss* ^*−/−*^ cells was able to partially recover over time, with differences seen to a greater extent at earlier time-points (Fig. 4a(iii)). This implies that the role CTSS plays in IL-1β maturation is an early onset effect and that at later time-points other factors may be able to compensate. Finally, we then incubated wild type and *ctss* ^*−/−*^ macrophages with alum, silica or polystyrene particulates and again observed significantly diminished IL-1β release in cells lacking CTSS, in both peritoneal and BMDMs (Fig. 4b and c). Taken together, these results indicate that CTSS plays a key role in the release of IL-1β in macrophages upon particle-induced lysosomal disruption.

### Both production of pro-IL-1β and Caspase 1 activity is partially dependent on CTSS

**Fig. 5:**
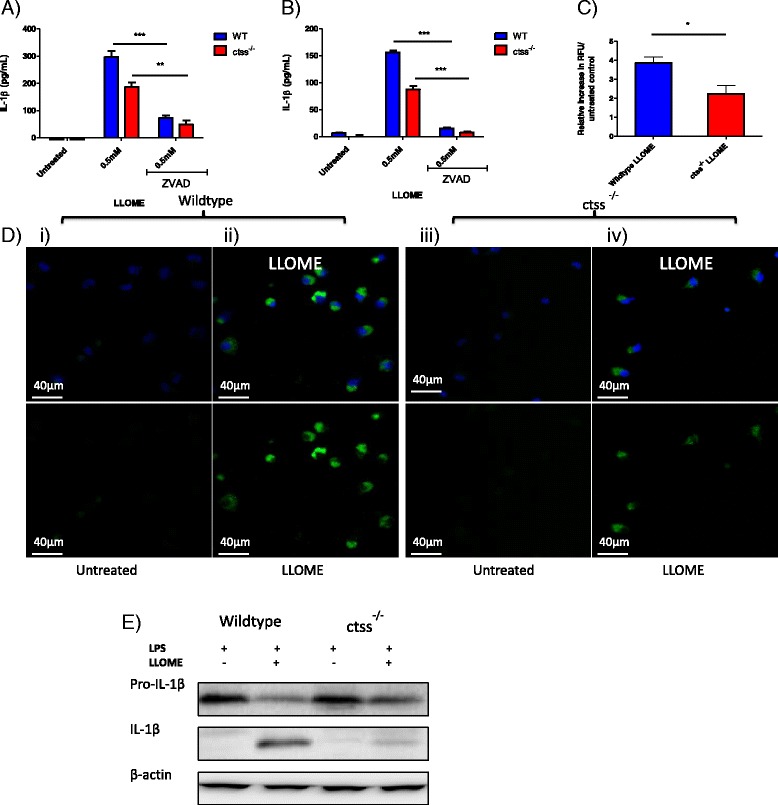
IL-1β processing in macrophages is partially dependent on CTSS. ELISA analysis of IL-1β release in supernatants from: **a** Wildtype and *ctss* ^*−/−*^ LPS-primed peritoneal macrophages and (**b**) Wildtype and *ctss* ^*−/−*^ LPS-primed BMDMs, which were pre-treated with Z-VAD-FMK (5uM) for 1 h prior to stimulation with LLOME (0.5 mM) for 16 h, *n* = 3 +/− standard error. **c** Caspase 1 activity measured in lysates generated from wildtype and *ctss* ^*−/−*^ BMDMs, untreated or treated with LLOME (0.5 mM) for 2 h. Activity was measured by RFU generated from caspase 1 mediated hydrolysis of Z-YVAD-AMC (50 μM). Data presented as increased RFU in lysates from treated cells relative to untreated control, *n* = 5 +/− error. **d** Confocal microscopy of active caspase in wildtype (WT) and *ctss* ^*−/−*^ peritoneal macrophages, treated for 2 h with LLOME (0.5 mM). Cells were incubated for 1 h with FAM-YVAD-FMK prior to washing, fixing and nuclear staining. Top panel: FLICA probe (*green*) + dapi (*blue*). Bottom panel: FLICA probe only (*green*). Images representative of 3 independent experiments, scale bar = 40 μM. **e** Western blot of the pro-form and active form of IL-1β in lysates generated from wildtype and *ctss* ^*−/−*^ LPS-primed BMDMs treated with LLOME (0.5 mM) for 5 h, before being harvested, lysed and blotted for IL-1β

Although we had established a role for CTSS in the release of IL-1β, the absence of CTSS did not have a complete inhibitory effect on the release of this cytokine. Therefore, the effect of CTSS knockdown in combination with caspase inhibition was examined. Here we found that in the *ctss* ^*−/−*^ macrophages, caspase inhibition still reduced IL-1β levels in both peritoneal macrophages and BMDMs (Fig. 5a and b). Next we compared intracellular caspase 1 activity in peritoneal wild-type and *ctss* ^*−/−*^ macrophages using the reporter substrate, Z-Tyr-Val-Ala-Asp-AMC, and the caspase 1 ABP used previously. At 2 h post LLOME treatment, marked caspase 1 activity was observed in both wildtype and *ctss* ^*−/−*^ cells, as seen by increased substrate turnover (Fig. 5c) and increased cytoplasmic green fluorescence from the ABP (Fig. 5d). However, consistent with release of IL-1β, there appeared to be reduced caspase 1 activity in the *ctss* ^*−/−*^ cells compared to wildtype cells. Taken together, this suggests that under these conditions in both peritoneal macrophages and BMDMs, the maturation of IL-1β is caspase 1 dependent, as expected, but that caspase 1 activity is modulated significantly by CTSS, consistent with its release during lysosomal disruption.

It has been shown that components of the inflammasome, pro-caspase 1 and pro-IL-1β can be secreted from macrophages upon treatment with cytoplasmic RNA, ATP or nigericin [27, 28]. Therefore, having observed that CTSS may contribute to the activation of caspase 1 following lysosomal rupture, we wanted to establish if the effect of CTSS on caspase 1-dependent IL-1β maturation was occurring inside or outside the cell. Western blot analysis of lysates from BMDMs revealed that LLOME causes the appearance of mature IL-1β; which was markedly abrogated in *ctss* ^*−/−*^ cells (Fig. 5e). This suggests that the role of CTSS in caspase 1-dependent processing of IL-1β is intracellular under these assay conditions. Furthermore, no extracellular caspase activity was detected using the fluorogenic substrate Z-Tyr-Val-Ala-Asp-AMC (Additional file 1: Figure S2). This further implies that the action of CTSS on caspase 1-dependent IL-1β maturation is intracellular and also confirms that the secretion of CTSS is specific and not merely due to cell membrane permeabilisation occurring during pyroptosis [29].

## Discussion

Concerns regarding the potential of nanotherapeutics to induce unwanted inflammatory and toxic side effects in vivo are supported by studies which have shown that particulates, such as alum, silica and monosodium urate, can activate NLRP3 signaling and IL-1β release [8, 13, 15]. Notably, overproduction of IL-1β has been linked causatively to silicosis [30, 31] and reduction of silicosis in vivo has been observed upon treatment with an IL-1 receptor antagonist, as well as in IL-1β knockout mice; suggesting that IL-1β is a key mediator of particle-induced inflammation [32]. While IL-1β levels can be easily quantified, with the rapidly growing field of proteolytic imaging permitting real time, non-invasive in vivo imaging of intact animal models or ex vivo imaging of patient samples, proteolytic enzymes may prove more attractive prognostic and diagnostic biomarkers. The identification of such biomarkers may be of value during both preclinical and clinical stages of the development of nanotherapeutics and also in understanding the molecular basis of adjunvanticity and particle-induced inflammation.

In this study we have demonstrated that, upon lysosomal rupture, a detectable increase in intracellular caspase 1 activity can be measured using peptidyl substrates and ABPs, which is in positive correlation with pro-inflammatory IL-1β release. Furthermore, we have shown that active CTSS is also released extracellularly upon lysosomal rupture. This data validates the utility of using protease activity signatures as rapid readouts of particle-induced lysosomal disruption in macrophage populations. Moreover, we have also identified CTSS as a critical factor in LPS-primed macrophages during production and processing IL-1β, induced by both LLOME and particulates.

Initially, the potential of particulates, composed of different materials, to activate IL-1β responses in LPS-primed murine macrophages was evaluated. While particles composed of the biodegradable polymer, PLGA, had no effect under these conditions, other non-biodegradable particles perturbed lysosomal integrity and generated IL-1β in a dose-dependent fashion, suggesting that these particulates were acting similarly in these experiments. Subsequently, we have demonstrated that direct lysosomal disruption with LLOME was able to mimic the IL-1β release profile induced by particulates, consistent with reports that accumulation of insoluble particulates can cause lysosomal disruption [13–15]. Using this model system, the potential roles of upstream mediators were further explored in order to identify biomarkers of particle-induced inflammation that could be applicable for in vivo imaging.

As IL-1β maturation has been shown to be dependent on both caspase 1 and lysosomal cathepsins, we sought to utilize the activities of these enzymes as readouts of toxicity. Firstly, using a fluorescently labelled affinity-based probe, FAM-YVAD-FMK, levels of intracellular caspase 1 were directly visualised in macrophages by fluorescent microscopy. Upon treatment with LLOME, caspase 1 activation was detected as early as 0.5 h and increased at 2 hours. Activity levels correlated with IL-1β release, demonstrating this protease could be used as a real-time biomarker of lysosomal rupture.

Next, we examined the potential to use lysosomal cathepsins in a similar capacity as signatures of lysosomal disruption, focusing on CTSS. CTSS is a lysosomal cysteine protease which is highly expressed in macrophages [33], has specific roles in MHC class II antigen presentation [34], toll-like receptor signaling [35] and is implicated in the pathology of multiple immune disorders, including rheumatoid arthritis, emphysema and atherosclerosis [36–39]. Notably, CTSS can retain activity at a neutral pH, outside the lysosomal pathway and also has limited tissue expression compared to other ubiquitously expressed cathepsins, meaning it could potentially give a more specific signal, with less background if imaged using ABP technology. [23, 33].

We demonstrate herein that the selection of an appropriate coumarin-linked peptidyl substrate coupled with appropriate assay conditions to favour the more stable CTSS over other members of the cysteine cathepsins, we were able to accurately detect and measure CTSS activity. Using these assay conditions it was observed that lysosomal destabilization with LLOME resulted in a decrease of CTSS activity in total lysates at 2 h. A similar finding has been reported previously with respect to a decrease in lysosomal cathepsin B activity upon treatment of macrophages with silica [13], which may be a consequence of the active protease species being quenched by cystatin inhibitors, naturally found in the cytoplasm [40]. Crucially, however, was the unanticipated effect that lysosomal disruption had on secretion of CTSS, where an increase in secreted CTSS activity was observed, using both substrate and ABP approaches, following LLOME-induced lysosomal rupture. Whilst extracellular expression of cathepsins has been well documented, particularly in inflammatory and neoplastic conditions [16], it was surprising to observe increased extracellular CTSS activity induced by lysosomal disruption. While cathepsin B may also be secreted, this protease requires the reducing, slightly acidic conditions of the lysosomes to facilitate activity, unlike CTSS. The molecular mechanisms underpinning cathepsin secretion are poorly understood, nonetheless these results reveal its utility as a potential extracellular biomarker of lysosomal disruption.

These investigations also permitted investigation of the possible relationship between CTSS and caspase 1 following lysosomal rupture. Cathepsins B, C and D have been previously implicated at various stages of IL-1β release from macrophages, dendritic cells and microglial cells, following treatment with a range of inflammatory stimuli [13, 41–43]. In keeping with these previous observations, inhibition of lysosomal acidification and use of a broad spectrum cysteine cathepsin inhibitor attenuated IL-1β release. However, using *ctss* ^*−/−*^ macrophages we identified CTSS as a major contributing factor for IL-1β release in these studies.

Caspase 1 activation in the *ctss* ^*−/−*^ background was subsequently examined, observing diminished activation, consistent with CTSS modulating IL-1β release through caspase 1.While we have shown that intracellular CTSS levels decrease following lysosomal rupture, there is likely to be a window of activation before CTSS becomes inhibited by cystatins or exported from the cell. This is supported by our observation that caspase 1 is active at 0.5 h post-LLOME treatment and lysates generated from LLOME-treated cells at this time retained CTSS activity, compared to a 2 hour time-point. However, it is noteworthy that the reduction in caspase activity in stimulated *ctss* ^*−/−*^ macrophages is only a partial effect, with approximately 25 % less caspase 1 activity than stimulated wildtype macrophages, suggesting CTSS may have other roles in the IL-1β response, besides contributing to caspase-1 activation.

In keeping with our reported findings, several studies have demonstrated that for caspase 1-mediated processing and secretion of mature IL-1β, particle-induced lysosomal rupture and subsequent cathepsin activity are required [11, 13, 44]. It has also recently been reported that following stimulation with alum, CTSS is crucial for IL-1β release from dendritic cells both in vitro and in vivo at the site of alum injection [45].

Interestingly, this was shown to be an inflammasome-independent process, potentially implying an alternative role for CTSS in the unconventional secretion of IL-1β. Therefore, we suggest a hypothetical model whereby CTSS may regulate multiple steps in the IL-1β response, potentially playing a direct role in modulating caspase 1-mediated processing of pro-IL-1β and also in unconventional protein release pathways. It may also be possible that the cytoplasmic presence of CTSS may be sensed as a pro-inflammatory danger signal and clearly further work is needed to dissect these speculative mechanisms.

Our findings that residual IL-1β was still detected in the *ctss* ^*−/−*^ cells following lysosomal rupture, confirmed the potential redundancy of cathepsins in the IL-1β response. Time course studies implied that CTSS has an early onset role IL-1β maturation, as at later time-points the reduction of IL-1β in *ctss* ^*−/−*^ cells becomes less significant, perhaps due to the eventual up-regulation of other cathepsins as a compensatory effect. Importantly, the broad spectrum inactivation of lysosomal cathepsins, with E64d and bafilomycin, attenuated release of IL-1β and caspase 1 activity to a greater extent than that seen in the *ctss* ^*−/−*^ macrophages, indicating the involvement of other proteases, such as cathepsin B, which is supported by a recent study exploring the redundancy of various cathepsins in inflammasome activation and the IL-1β response [44].

## Conclusion

Taken together, we have demonstrated that extracellular CTSS activity levels and intracellular caspase 1 activity levels can act as surrogate biomarkers for lysosomal disruption and particle-induced inflammation in vitro. Furthermore, given recent reports that CTSS can be detected in serum and bronchoalveolar lavage fluid, CTSS may prove to be a meaningful clinical biomarker of particle-induced inflammation. There are already excellent reagents for the determination of caspase 1 activity and although historically it has been challenging to develop selective reagents towards CTSS, many recent studies have highlighted new generation ABPs and substrates that may overcome such bottlenecks [46, 47], and indeed already applied to assess CTSS activity levels in the serum of rheumatoid arthritis and osteoarthritis patients to stratify disease states [48]. In the future, the pre-clinical and clinical application of such techniques in assessing the real time activities of caspase 1 and CTSS could prove extremely useful in determining acute particulate-induced inflammation during the development of nanotherapeutics.

## Methods

### Cell isolation and culture

Primary cells used in the following experiments were extracted from aged-matched C57BL/6 mice and CTSS deficient mice (*ctss* ^*−/−*^) subsequent to euthanasia by CO_2_. C57BL/6 mice were purchased from Charles Rivers Laboratories and *ctss* ^*−/−*^ mice were obtained from J. A. Joyce, Memorial Sloan Kettering Cancer Center, New York. Animal handling, housing and primary cell isolation was carried out in accordance with the UK Home Office and approved by Queen's University Belfast Ethical Review Committee.

Peritoneal macrophages were isolated through injection of sterile PBS into the peritoneal cavity. Cells were washed is PBS, counted, seeded and left to adhere for at least 2 hours prior to treatments. Cells were then cultured in DMEM medium which was supplemented with 10 % low endotoxin fetal calf serum and 1 % penicillin/streptomycin, in a 37 °C, 5 % CO_2_ humidified incubator. Bone marrow derived macrophages were generated from flushing the leg bones of C57BL/6 mice and *ctss* ^*−/−*^ mice to isolate bone marrow. Bone marrow cells were then cultured for 1 week in DMEM supplemented with 30 % L929 supernatant, containing granulocyte-macrophage colony-stimulating factor (GM-CSF), to drive differentiation.

To induce expression of pro- IL-1β, all cells were primed for 3 h with toll-like receptor ligand *Escherichia coli* R515 LPS (100 ng/mL) (InvivoGen) prior to treatments with particulates or LLOME (Sigma). PSNP and silica nanoparticles were 100 nm in diameter and positively charged and were purchased from Micromod in 25 mg/mL suspensions. Alhydrogel adjuvant, referred to as ‘alum’ throughout this manuscript, was purchased from Invivogen in a 10 mg/mL suspension and particles were an average of 10 μm in diameter and of neutral charge. Blank PLGA nanoparticles, 150 nm in diameter and of neutral zeta potential were prepared as described in [49]. Cells were treated for the time-points stated and where indicated, cells were pre-incubated for 1 h with 50 μM E64d (Enzo Life Sciences), 250 nM bafilomycin A1 (Sigma) or 5 μM Z-VAD-FMK (Calbiochem).

### ELISA

Cell culture supernatants were assayed for IL-1β secretion using an R&D DuoSet ELISA (DY401), according to manufacturer’s instructions.

### Confocal microscopy

Following treatments, living cells were incubated for 1 h with caspase 1 probe, FAM-YVAD-FMK, according to manufacturer’s instructions (Immunochemistry Technologies). Alternatively, cells were incubated for 0.5 h with 2.5 μM acridine orange (Thermofischer Scientific) to label acidic lysosomal compartments. After being washed in PBS twice, cells were fixed in 4 % Paraformaldehyde in PBS for 20 mins, mounted with ProLong Gold Antifade Mountant containing DAPI (Invitrogen) and visualised using a Nikon TE EZ-C1 confocal system (Nikon, Kingston Upon Thames). Images were captured with a 60 × objective lens and a 1024 × 1024 for the caspase 1 probe and a 512 × 512 frame for the lysosomal staining.

### Caspase 1 activity assay

Following treatments, BMDMs were lysed in Hepes lysis buffer (25 mM Hepes, 100 mM NaCl, 2 nM EDTA, 0.1 % CHAPs, 10 % sucrose, pH 7.4) according to Thornberry et al., [6]. Lysates (80 μg) were incubated with fluorogenic substrate, Z-YVAD-AMC (50 μM) (Enzo Life Sciences) to measure caspase 1 activity in activity buffer (50 mM Hepes, 10 % sucrose, 1 % CHAPS. 1 mM EDTA, 10 mM DTT, 1 mM PMSF, 10 μM E64, 10 μM pepstatin A). Substrate turnover was measured after 1 h at excitation/emission 385 nm/460 nm.

### Cathepsin S activity assay

Following treatments, BMDMs were harvested and lysed in sodium acetate lysis buffer (100 mM NaOAc, 100 mM NaCl, 0.1 % Triton X-100, pH 5.5) by incubation on ice for 0.5 h, followed by pulse sonication. Lysates were then clarified by centrifugation at 14,000 x *g* for 10 mins at 4 °C. Lysates were then incubated with sodium phosphate buffer (0.1 M Na_2_HPO_4_, 0.1 M NA_2_H_2_PO_4_, pH 7.4) for 45 mins to inactivate other cathepsins. At this point lysate mixtures or cell supernatants were added to MES reaction buffer (0.5 M MES, 1 mM EDTA, 2 mM DTT, pH 6) and 50 μM Z-VVR-AMC (Enzo Life Sciences) was then added. Substrate turnover was measured after 1 h at excitation/emission 385 nm/460 nm.

### Cathepsin S ABP labelling

Supernatants from BMDMs that had been treated with LLOME for the indicated time-points were diluted in MES reaction buffer and incubated at 37 °C for 15 mins with 10 μM biotin-PEG-LVG-DMK. The reaction was terminated by addition of 5X Laemmli buffer and denaturation at 95 °C. Samples were resolved by 15 % SDS-PAGE and transferred onto nitrocellulose PVDF membranes (Millipore), blots were subsequently blocked in 5 % BSA in TBS/tween overnight at 4 °C. The membrane was then incubated with Streptavidin-horse radish peroxidase (Cell Signaling) diluted TBS/Tween, with 5 % BSA for 0.5 h at room temperature. Proteins were imaged using ECL Plus chemiluminescent substrate (ThermoFisher Scientific) and exposed using the ChemiDoc XRS imaging system (BioRad Laboratories).

### Immunoblot analysis

Lysates generated from BMDMs, were separated by 15 % SDS-PAGE and transferred onto nitrocellulose PVDF membranes (Millipore). Blots were immersed in 5 % milk powder in TBS/tween and incubated overnight at 4 °C with a rat monoclonal antibody for IL-1β (RnD Systems AF-501). Membranes were then washed and incubated with rabbit anti-rat secondary antibody (Abcam). Proteins were imaged using ECL Plus chemi luminescent substrate (ThermoFisher Scientific) and exposed using the ChemiDoc XRS imaging system (BioRad Laboratories).

### Statistical analysis

Data was analyzed for statistical significance using GraphPad software. The experimental values are shown as the means +/− standard error. An unpaired t-test was used for all analyses and a *P*-value of <0.05 was considered to indicate statistical significance.

## Additional file

Additional file 1: Figure S1. PLGA does not induce release of IL-1β in peritoneal macrophages. ELISA analysis of IL-1β production in supernatants peritoneal macrophages which were primed for 3h with LPS (100ng/ml) and stimulated for 16h with a concentration range of PLGA. **Figure S2.** LLOME does not induce extracellular caspase1 activity. Caspase 1 activity in supernatants from LPS-primed BMDMs, treated with LLOME (0.5mM) for 45 mins to 6h. Activity was measured by RFU, generated from caspase 1 mediated hydrolysis of Z-YVAD-AMC. (PPTX 56 kb)

## Abbreviations

ABPs: affinity-binding probes
AMC-7: amino-4-methylcoumarin
BMDMs: bone marrow derived macrophages
CTSS: cathepsin S
IL-1β: interleuekin-1 beta
LLOME: L-leucyl-L-leucine methyl ester
LPS: lipopolysaccharide
PLGA: poly-lactide-co-glycolic acid
PSNP: polystyrene nanoparticulates
