# Supplementary figures and images for: Extracellular cathepsin S and intracellular caspase 1 activation are surrogate biomarkers of particulate-induced lysosomal disruption in macrophages
Source: Part Fibre Toxicol. 2016 Apr 23;13:19. doi: 10.1186/s12989-016-0129-5 (PMC4842290; doi:10.1186/s12989-016-0129-5)

## Slide 1
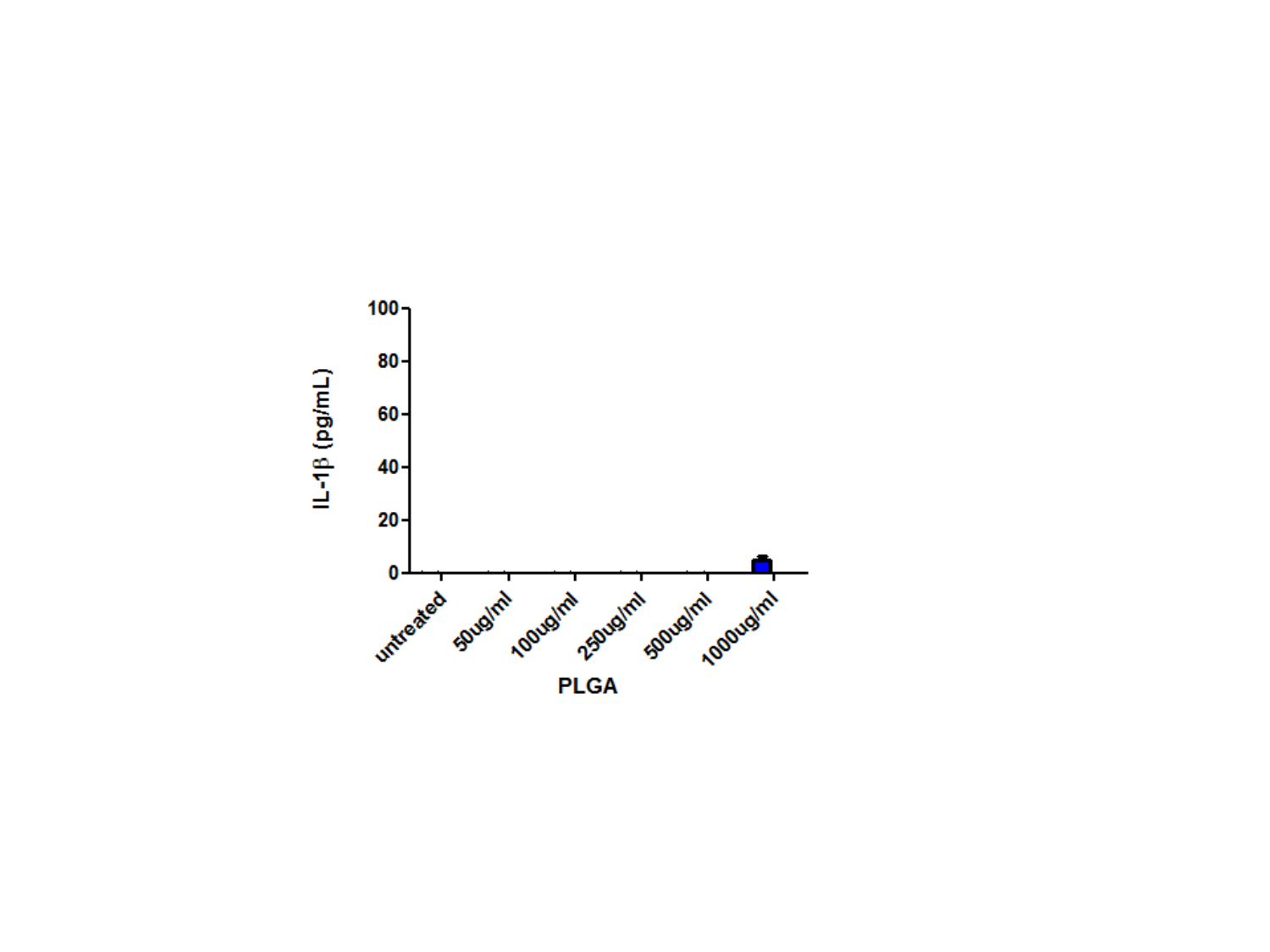

## Slide 2
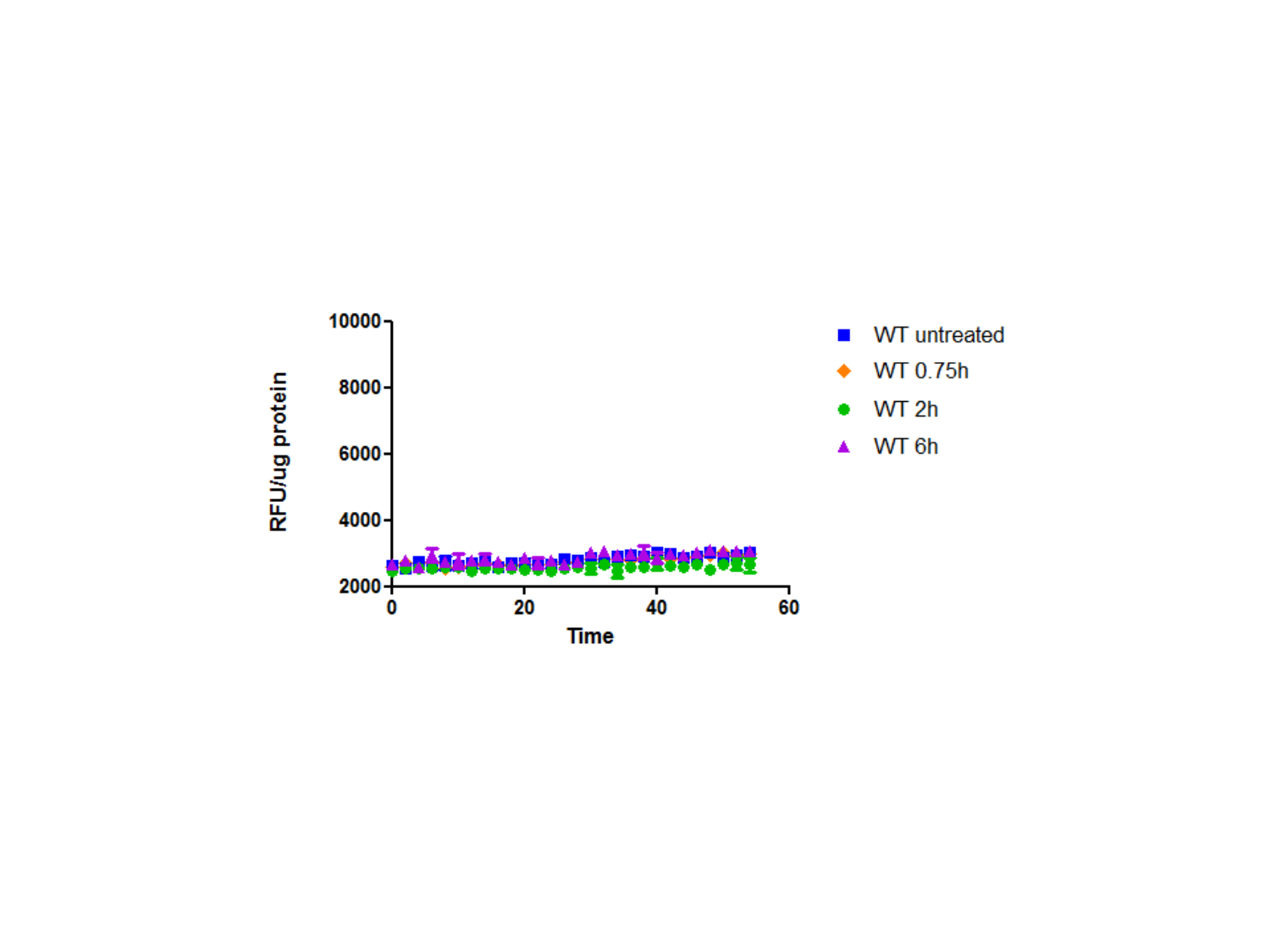

Supplement: Additional file 1: Figure S1. — PLGA does not induce release of IL-1β in peritoneal macrophages. ELISA analysis of IL-1β production in supernatants peritoneal macrophages which were primed for 3h with LPS (100ng/ml) and stimulated for 16h with a concentration range of PLGA. Figure S2. LLOME does not induce extracellular caspase1 activity. Caspase 1 activity in supernatants from LPS-primed BMDMs, treated with LLOME (0.5mM) for 45 mins to 6h. Activity was measured by RFU, generated from caspase 1 mediated hydrolysis of Z-YVAD-AMC. (PPTX 56 kb) [file 12989_2016_129_MOESM1_ESM.pptx]
